# An Evaluation of Novel Inflammatory Biomarkers in Children with *Kingella kingae* Osteoarticular Infections

**DOI:** 10.3390/pathogens15070763

**Published:** 2026-07-20

**Authors:** Matteo Fortunato, Anne Tabard-Fougère, Giacomo De Marco, Oscar Vazquez, Christina Steiger, Elio Paris, Ardian Ramadani, Andreas Tsoupras, Romain Dayer, Dimitri Ceroni

**Affiliations:** 1 Faculty of Medicine, University of Geneva, 1206 Geneva, Switzerland; matteo.fortunato@etu.unige.ch (M.F.);; 2 Paediatric Orthopaedics Service, Geneva Children’s Hospital, Geneva University Hospitals, 1211 Geneva, Switzerland

**Keywords:** osteoarticular infections, *Kingella kingae*, immune-inflammatory biomarkers

## Abstract

*Kingella kingae* is now recognised as the leading cause of osteoarticular infections (OAIs) in young children. Because these infections typically produce mild symptoms and a weak inflammatory response, early diagnosis remains challenging. Complete blood count-derived immune-inflammatory biomarkers (IIBs) have recently gained interest as accessible, low-cost indicators of systemic inflammation, with potential diagnostic value in several fields, including oncology, rheumatology, cardiology, and infectious diseases. This study therefore aimed to assess whether IIBs could support the screening of *K. kingae* OAIs. We retrospectively reviewed the medical records of 209 children admitted to our hospital between 2007 and 2025 with confirmed or highly suspected *K. kingae* OAIs. Complete blood counts were analysed for each patient, including leukocyte subtypes such as neutrophils, lymphocytes, monocytes, eosinophils, and basophils. We then calculated biomarkers (NLR, MLR, PLR, SII, SIRI, and PIV) and interpreted them using age-adjusted reference values. As expected, most children were younger than 48 months, and septic arthritis was the predominant clinical presentation. Classical acute-phase reactants (WBC, CRP, and ESR) were frequently normal or only mildly elevated. Likewise, most IIBs remained within or near reference ranges; the platelet-to-lymphocyte ratio was the most commonly abnormal marker, exceeding the threshold in only 59.3% of patients. These results indicate that, like conventional inflammatory markers, CBC-derived IIBs have limited standalone screening utility for *K. kingae* OAIs. Further studies are needed to determine whether systemic IIBs can help distinguish OAIs caused by *K. kingae* from those caused by pyogenic bacteria.

## 1. Introduction

Since 1988, Pablo Yagupsky has emphasised the important role of *Kingella kingae* (*K. kingae*) in osteoarticular infections (OAIs) in infants and toddlers. Since then, reported cases of *K. kingae*-related OAIs have risen sharply, largely because of advances in molecular diagnostic techniques [1,2,3,4,5,6,7]. Numerous studies now identify *K. kingae* as the leading bacterial cause of OAIs in children aged 6 to 48 months, accounting for 30–93.8% of culture-positive cases [1,2,3,4,8,9,10]. Beyond septic arthritis and acute hematogenous osteomyelitis, this pathogen can also cause less typical infections such as spondylodiscitis [11,12,13,14], subacute osteomyelitis [15,16], pyomyositis [17], bursitis [18], and tendon sheath infections [19]. Regardless of the infection site, *K. kingae* OAIs typically cause only mild symptoms and a limited inflammatory response, making them difficult to identify [20]. In fact, only 10–33% of affected children have a temperature ≥38 °C at admission [1,2,3,10,21], and most show normal or near-normal white blood cell counts and C-reactive protein levels [1,2,10,21]. In contrast, erythrocyte sedimentation rate and platelet count appear to be the most sensitive conventional inflammatory markers in *K. kingae* OAI [1,10]. Despite these observations, the diagnosis of *K. kingae* OAIs remains challenging, and research over the past two decades has concentrated on improving their detection through blood tests with modest success.

Over the past decade, complete blood count (CBC)-derived immune–inflammatory biomarkers (IIBs), including neutrophil-to-lymphocyte ratio (NLR), monocyte-to-lymphocyte ratio (MLR), platelet-to-lymphocyte ratio (PLR), systemic immune–inflammation index (SII), systemic inflammatory response index (SIRI), and pan-immune–inflammation value (PIV), have gained increasing attention as accessible markers for predicting disease progression and prognosis in numerous clinical contexts [22]. Their potential use has been explored throughout the continuum of patient care, including risk prediction, diagnosis, treatment selection, disease monitoring, and prognostic assessment, particularly in fields such as oncology, rheumatology, infectious diseases, and cardiology [22,23,24,25,26,27,28,29,30]. In OAIs, their association with systemic inflammation has likewise supported interest in IIBs. Nevertheless, the available evidence for IIBs in paediatric OAIs remains limited to selected clinical settings, relies predominantly on adult populations or mixed bacterial aetiologies, and has seldom addressed whether these biomarkers can assist in identifying specific causative pathogens. It has not specifically addressed *K. kingae* infections, which are characterised by a distinctly mild inflammatory profile.

Accordingly, this study aimed to characterise CBC-derived IIBs in a large cohort of children with *K. kingae* osteoarticular infections (OAIs) and to assess their standalone screening value by comparing observed values to age-adjusted reference thresholds derived from physiologic blood cell count.

## 2. Materials and Methods

### 2.1. Study Design and Setting

Following approval by the Children’s Hospital Ethics Review Committee (2023-00578), we conducted a retrospective cohort study of all patients admitted to the Geneva University Hospitals, a tertiary paediatric referral centre, with confirmed and suspected *K. kingae*-related OAI between January 2007 and December 2025. January 2007 was chosen as the study start date because it marked the introduction of molecular diagnostic methods for *K. kingae* at our hospital.

### 2.2. Population and Case Definitions

*K. kingae* infections were classified as confirmed when culture or PCR tests of blood samples and/or biopsy samples from infected sites were positive, together with positive MRI findings or standard radiographs. Patients with highly suggestive clinical and biological features, compatible imaging, and a positive oropharyngeal swab for *K. kingae* were also included and classified as highly suspected *K. kingae* cases.

### 2.3. Data Collection

For each patient, age at diagnosis, gender, type of infection, and locations were extracted from medical records. Thus, patients were classified as having septic arthritis (SA), acute or subacute hematogenous osteomyelitis, arthritis with concomitant osteomyelitis, spondylodiscitis, or another form of OAI (including pyomyositis and tenosynovitis).

The blood investigations collected at admission included the erythrocyte sedimentation rate (ESR), C-reactive protein value (CRP), white blood cell (WBC) count, and neutrophil (G/L), lymphocyte (G/L), monocyte (G/L), and platelet counts.

With the above-cited parameters, six CBC-derived IIB markers were calculated using these formulas:

- NLR = neutrophils/lymphocytes;
- MLR = monocytes/lymphocytes;
- PLR = platelets/lymphocytes;
- SII = platelets × neutrophil/lymphocyte ratio;
- SIRI = neutrophils × monocyte/lymphocyte ratio;
- PIV = neutrophils × monocytes × platelet/lymphocyte ratio (PIV).

All calculations were performed consistently using cell counts expressed as G/L. NLR, MLR, and PLR were treated as dimensionless ratios. SII, SIRI, and PIV were treated as scale-dependent composite indices rather than dimensionless ratios.

### 2.4. Age-Adjusted Reference Values

Reference values for these IIBs appeared to vary substantially with age, sex, and other factors. However, most available data come from adult cohorts, frequently including patients with underlying conditions, while paediatric reference data remain limited [31,32,33,34,35,36,37]. Thus, we tried to establish baseline values for these IIBs for children aged 0–48 by using the normative values in accordance with our hospital’s guidelines [38,39,40]. For each blood-cell parameter, only the upper reference limit was retained. These upper reference limits were then used to derive estimated normative thresholds for NLR, MLR, PLR, SII, SIRI, and PIV. The same thresholds were applied to all patients in the cohort.

### 2.5. Microbiological Investigations

Identification of the causative microorganism was systematically attempted using blood cultures. Before 2009, BACTEC 9000 blood culture media were used (Becton Dickinson, Eysins, Switzerland); thereafter, cultures were processed with the automated BD BACTEC FX system (Becton Dickinson). Specimens such as joint fluid or bone aspirate were sent to the laboratory for Gram staining, cell counting, and immediate inoculation onto Columbia blood agar and CDC anaerobe 5% sheep blood agar, both incubated under anaerobic conditions, as well as onto chocolate agar incubated in a CO_2_-enriched atmosphere and into brain–heart infusion broth. All culture media were incubated for 10 days. From 2007 onward, a real-time PCR assay targeting the RTX toxin genes of *K. kingae* was used [6]. This assay targets *rtxA* and *rtxB*, two independent genes within the RTX toxin locus [6], and was applied to various biological samples, including synovial fluid, bone, and peripheral blood. Since September 2009, oropharyngeal swab PCR has also been performed in children aged 6 months to 4 years. Detection of the RTX toxin genes in the oropharynx provides strong evidence supporting *K. kingae* as the cause of OAI, whereas their absence makes this diagnosis unlikely [41].

### 2.6. Statistical Analysis

All statistical analyses were performed using R software (version 4.4.1; R Foundation for Statistical Computing, Vienna, Austria) and the RStudio interface (version 2024.09.0; Posit). The level of significance was set at *p* < 0.05, and 95% confidence intervals (CIs) and effect sizes were calculated.

Continuous variables are presented as mean ± standard deviation or median (interquartile range) depending on the distribution. Categorical variables are presented as frequencies and percentages. The normality of continuous variables was assessed using quantile–quantile (Q-Q) plots and the Shapiro–Wilk test.

For each IIB, we calculated the proportion of patients with values above the age-adjusted upper reference limit. Subgroup comparisons between confirmed and highly suspected cases were performed using the Mann–Whitney U test for continuous variables and chi-square or Fisher’s exact test for categorical variables.

## 3. Results

### 3.1. Epidemiology and Skeletal Distribution

Of the 238 children treated at our hospital for confirmed (*n* = 141) or highly suspected (*n* = 97) *K. kingae* OAIs, 209 had complete blood count data available and were included in the analysis.

**Figure 1:**
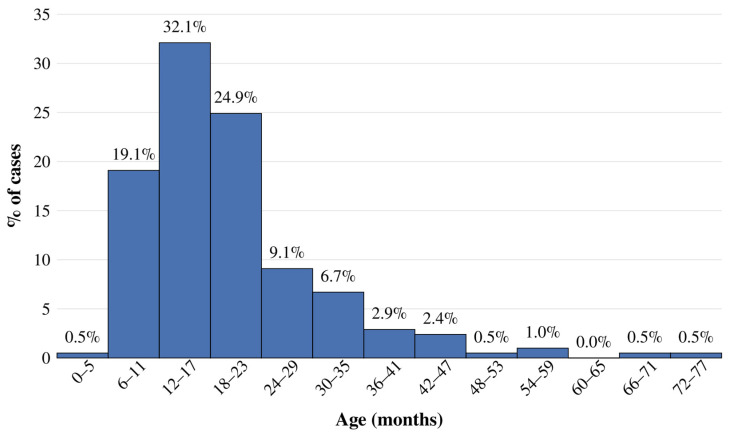
Age distribution of all cases.

The cohort comprised 100 girls and 109 boys, with a mean age of 19.7 ± 10.7 months (Table 1). No infections occurred in children younger than 3 months, and 204 patients (97.6%) were under 48 months at disease onset. *K. kingae* OAIs were most frequent in the 12-to-17-month age group, affecting 67 children (32.1%). The age distribution of the cohort is shown in Figure 1. Septic arthritis was the most common presentation (98 cases), followed by osteomyelitis (46 cases), arthritis with concomitant osteomyelitis (22 cases), tenosynovitis, and spondylodiscitis (19 cases each). Among septic arthritis cases, the knee was the predominant site (54 cases), well ahead of the hip (11 cases), wrist (7 cases), and ankle (7 cases). Hematogenous osteomyelitis most often involved long bones (22 cases) and tarsal or carpal bones (19 cases), with fewer cases affecting flat bones (2 cases), the patella (1 case), the thorax (1 case), or the spine (1 case). The femur was the most involved long bone, accounting for 11 cases (50%). OAI types and anatomical sites are detailed in Table 1.

### 3.2. Bacteriological Investigations

Among the bacteriological investigations, blood and biopsy (joint sample and bone aspirate) cultures were positive in 6.8% (10 of 146) and 7.7% (11 of 142) of cases, respectively. PCR testing of blood and biopsy was positive in 109 of 141 cases (77.3%). The oropharyngeal swab for *K. kingae* RTX toxin genes had the highest percentage of positive cases, with 170 of 175 (97.1%) children testing positive.

### 3.3. Clinical and Inflammatory Markers

**Figure 2:**
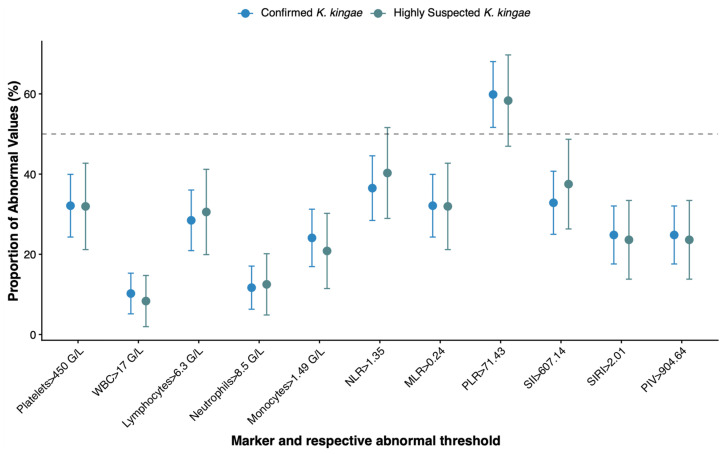
Proportion of markers above their respective age-adjusted reference values and 95% confidence intervals. Note: x-axis numbers refer to upper threshold values; WBC, white blood cell count; NLR, neutrophil-to-lymphocyte ratio; MLR, monocyte-to-lymphocyte ratio; PLR, platelet-to-lymphocyte ratio; SII, systemic immune–inflammation index; SIRI, systemic inflammation response index; PIV, pan-immune–inflammation value.

No significant differences in classical inflammatory markers were identified between highly suspected and confirmed *K. kingae* OAIs (see Appendix A, Table A1). Using age-appropriate cut-off values for children under 4 years of age (Table 2), WBC counts were normal (<17,000 cells/µL) in 189 cases (90.4%), with a mean of 12,263 ± 3622 cells/µL (range: 3800–26,000 cells/µL). CRP, available in 208 cases, was normal (<10 mg/L) in 75 cases (36.1%); among the remaining 133 cases, the mean value was 36.7 ± 26.6 mg/L. ESR, available in 171 cases, exceeded 20 mm/h in 132 children (77.2%), with a mean of 35.8 ± 19.7 mm/h (range: 2–102 mm/h). No significant differences in classical inflammatory markers were identified between highly suspected and confirmed *K. kingae* OAIs. The blood cell counts used to calculate the IIBs are presented in Table 2. Compared with the age-specific physiological ranges shown in Table 2, neutrophil, lymphocyte, monocyte, and platelet counts remained within expected limits. Platelets had the highest proportion of abnormal values, observed in 67 of 209 (32.1%) cases (see Figure 2).

### 3.4. CBC-Derived Immune-Inflammatory Biomarkers

Table 3 compares IIBs observed in our cohort with those calculated from the age-specific physiological values for the different blood cell lineages underlying the different IIB indices.

Figure 2 shows the proportion of patients exceeding the pathological cutoff for each IIB. Subgroup analysis between highly suspected and confirmed *K. kingae* OAIs revealed no significant difference in any of the immune–inflammation biomarkers (see Appendix A, Table A1).

Although mean values were higher than the calculated references for all IIBs, only PLR was above the normative threshold in more than half of the patients (Figure 2), with 59.3% (124 of 209 cases). Published IIB reference values are summarised in Table 4.

## 4. Discussion

*K. kingae* is now recognised as the main cause of osteoarticular infections in young children, yet its diagnosis remains challenging. These infections typically present with an absent or low-grade fever and only modest increases in acute-phase reactants. As a result, routine blood tests have so far offered limited screening utility, highlighting the need to determine whether CBC-derived IIBs may provide a specific biological signature of *K. kingae* OAIs. To our knowledge, this is the first study to specifically assess IIBs in paediatric *K. kingae* OAIs.

Our findings confirm that *K. kingae* OAIs predominantly affect children younger than 48 months and that septic arthritis accounts for nearly half of cases, in line with previous reports. They also reinforce the knee as the joint most involved. Finally, our results highlight the persistent diagnostic challenge posed by *K. kingae* OAIs, which usually trigger only a modest systemic inflammatory response [1,2,3,10,25,26].

Overall, the biomarkers studied showed limited standalone screening utility for detecting *K. kingae* OAIs, as most values remained normal or near normal despite active infection, and their wide variability further complicated interpretation. In fact, most IIB values were within or only marginally above reference ranges, which could initially imply diagnostic relevance. This apparent elevation should therefore be interpreted cautiously, as it likely results from skewed distributions in which a small number of extreme outliers inflated the mean values. The particularly broad ranges observed for SIRI and PIV indicate substantial variability, with maximum values far above the corresponding medians.

The PLR illustrates this issue clearly. It exceeded the threshold in 59.3% of cases, meaning that 40.7% of patients with confirmed or highly probable *K. kingae* OAIs had a normal PLR value. The other indices performed even less well, with NLR, MLR, SII, SIRI, and PIV abnormal in fewer than half of cases, thereby limiting their value for standalone screening or diagnosis. Thus, the weak inflammatory response that reduces the diagnostic performance of conventional markers, including WBC count, CRP, and ESR, also appears to limit these composite biomarkers.

The limited systemic inflammatory response observed in *K. kingae* osteoarticular infections may reflect its immune evasion mechanisms that distinguish this pathogen from pyogenic bacteria. *K. kingae* produces a polysaccharide capsule and exopolysaccharide (galactan) that function synergistically to evade host defences: the capsule interferes with neutrophil oxidative burst and binding, while the exopolysaccharide blocks phagocytosis and resists antimicrobial peptides [42,43]. Additionally, *K. kingae* expresses a factor H-binding protein that recruits human factor H, inhibiting complement-mediated killing and serving as the major determinant of serum resistance [44]. These virulence factors enable *K. kingae* to survive in the bloodstream and disseminate while triggering minimal innate immune activation [43]. This attenuated host response may help explain why conventional markers and CBC-derived immune–inflammatory biomarkers often remain normal or only mildly elevated despite active infection.

Interpretation of these biomarkers is further complicated because most available data derive from adult cohorts, frequently including patients with underlying conditions such as diabetes, obesity, or smoking history, rather than healthy children, making comparisons difficult [31,32,33,34,35,36,37]. The limited paediatric reference data that do exist show considerable heterogeneity and do not study all six biomarkers [31,37].

This study has several limitations that must be acknowledged. First, its retrospective design may have resulted in missed cases because of coding errors, and some patients had to be excluded due to incomplete blood test data. It also introduced variability in clinical and laboratory assessments, as blood samples and biopsies were collected at different intervals after symptom onset. Given the dynamic nature of inflammatory responses, this timing may have affected biomarker values. The inclusion of highly suspected *K. kingae* OAIs also introduces a risk of misclassification.

In addition, the study did not include comparator groups of children with pyogenic osteoarticular infections, noninfectious musculoskeletal conditions mimicking infection, or healthy controls. Consequently, the observed clinical and biological characteristics should be considered descriptive rather than diagnostic. Comparative prospective studies are required to determine whether these findings can reliably distinguish *K. kingae* infections from other conditions.

A further limitation is that the proposed paediatric IIB thresholds were mathematically derived from the upper reference limits of individual blood-cell counts for children aged 0–48 months, rather than established in a healthy paediatric reference population, and should therefore be considered exploratory, pending external validation.

Despite the sample size limitations for inferential analyses, the descriptive data provide useful insights into the behaviour of these biomarkers in *K. kingae* OAIs. Second, interpretation is constrained by the absence of validated normative reference values for immune–inflammatory biomarkers in healthy children younger than 4 years. Although age-adjusted values derived from neutrophil, lymphocyte, monocyte, and platelet counts provided a practical comparator, they cannot establish sensitivity, specificity, or diagnostic cut-offs. These findings should therefore be regarded as exploratory and may help guide future studies aimed at defining clinically relevant paediatric thresholds.

## 5. Conclusions

In this large cohort, complete blood count-derived immune–inflammatory biomarkers showed limited diagnostic value for identifying *K. kingae* OAIs. The mild inflammatory response typical of these infections appears to affect both conventional markers, such as CRP, ESR, and WBC count, and composite IIBs, which often remained normal or only slightly increased despite active disease. Although PLR was the most frequently abnormal biomarker, it exceeded the threshold in only 59.3% of patients; NLR, MLR, SII, SIRI, and PIV were abnormal in fewer than half of cases, limiting their usefulness for standalone screening or diagnosis. Thus, these findings suggest that these biomarkers have limited standalone screening utility in children already suspected of having *K. kingae* OAIs. Future comparative studies should establish validated paediatric reference intervals and test whether these markers can distinguish *K. kingae*-associated from non-*K. kingae*-associated OAIs or from non-infectious mimics.

## Figures and Tables

**Table 1:** Epidemiology of the cohort (*N* = 209).

Characteristic	*n* (%)
Diagnostic classification	
Highly suspected *K. kingae* infection	88 (42.1)
Confirmed *K. kingae* infection	121 (57.9)
Demographics	
Age mean ± SD (months)	19.7 ± 10.7
Male sex	109 (52.2)
Female sex	100 (47.8)
Type of OAI (n = 225)	
Septic arthritis (SA)	98 (43.6)
Knee	54 (55.1)
Hip	11 (11.2)
Ankle	7 (7.1)
Wrist	7 (7.1)
Elbow	6 (6.1)
Other joints	13 (13.3)
Osteomyelitis (OM)	46 (20.4)
Long bones	22 (47.8)
Tarsal/carpal bones	19 (41.3)
Other bones	5 (10.9)
Arthritis with concomitant OM	22 (9.8)
Spondylodiscitis	19 (8.4)
Tenosynovitis	19 (8.4)
Other	21 (9.3)

Note: Percentages for diagnostic classification and demographics are based on *N* = 209. OAI categories are not mutually exclusive; 225 infection categories were recorded, and percentages for OAI type therefore use *n* = 225. Percentages for anatomical sites are calculated within the corresponding OAI category.

**Table 2:** Classical inflammatory markers and upper reference interval.

Marker	*n*	Mean ± SD	Median	Range	Upper Threshold
Abnormal CRP	133	36.72 ± 26.6	28.5	10.0–138.0	<10
ESR	171	35.8 ± 19.7	34.0	2.0–102.0	<20
WBC count	209	12.3 ± 3.6	11.9	3.8–26.0	<17
Neutrophils	209	5.5 ± 2.6	5.0	1.2–13.3	<8.5
Lymphocytes	209	5.1 ± 2.5	5.3	0.2–18.5	<6.3
Monocytes	209	1.4 ± 1.8	0.9	0.1–14.6	<1.49
Platelets	209	408 ± 121	383.5	123–824	<450

Note: SD, standard deviation; CRP, C-reactive protein in mg/L; ESR, erythrocyte sedimentation rate in mm/h; WBC, white blood cell count; all cell counts in ×10^9^/L. CRP was available in 208 cases and abnormal in 133 cases; normal CRP values were excluded from calculations. ESR was available in 171 cases.

**Table 3:** IIB reference values and cohort mean, standard deviation, median, and range.

Parameter	Estimated Threshold	Mean ± SD	Median	Range
NLR	1.35	1.96 ± 2.84	1.02	0.17–22.00
MLR	0.24	1.00 ± 3.16	0.18	0.02–26.50
PLR	71.43	134.03 ± 175.26	79.86	13.12–1290.63
SII	607.14	767.02 ± 1066.45	415.33	51.84–9086.00
SIRI	2.01	5.94 ± 19.35	0.88	0.05–186.56
PIV	904.64	2386.60 ± 8198.26	314.39	15.71–77,049.28

Note: SD, standard deviation; NLR, neutrophil-to-lymphocyte ratio; MLR, monocyte-to-lymphocyte ratio; PLR, platelet-to-lymphocyte ratio; SII, systemic immune–inflammation index; SIRI, systemic inflammation response index; PIV, pan-immune–inflammation value.

**Table 4:** IIB literature reference values.

Reference	NLR	MLR	PLR	SII	SIRI	PIV
Lee et al. [36]	0.29–2.98	0.12–0.56	62.32–195.63			
Fest et al. [37]	0.83–3.92		61–239	189–1168		
Meng et al. [38]	0.88–3.00	0.11–0.34	61–179	161–710		
Liu et al. [39]	0.88–4.00	0.1–0.37	49–198	142–804		
Amezcua-Guerra [40]						65.8–491.6
Karpuzoğlu et al. [41]	1.02–2.57				0.39–1.24	
Moosmann et al. [42]	0.51–3.02	0.10–0.71	50.07–191.24			

Note: NLR, neutrophil-to-lymphocyte ratio; MLR, monocyte-to-lymphocyte ratio; PLR, platelet-to-lymphocyte ratio; SII, systemic immune–inflammation index; SIRI, systemic inflammation response index; PIV, pan-immune–inflammation value.

## Data Availability

The data supporting the findings of this study are not openly available due to sensitivity concerns. They are accessible from the corresponding author upon reasonable request. The data are stored in a controlled-access repository at Geneva University Hospitals, Switzerland.

## Abbreviations

The following abbreviations are used in this manuscript:

OAI	Osteoarticular infections
IIB	Immune–inflammation biomarker
PCR	Polymerase chain reaction
CRP	C-reactive protein
ESR	Erythrocyte sedimentation rate
WBC	White blood cell count
NLR	Neutrophil-to-lymphocyte ratio
MLR	Monocyte-to-lymphocyte ratio
PLR	Platelet-to-lymphocyte ratio
SII	Systemic immune–inflammation index
SIRI	Systemic inflammation response index
PIV	Pan-immune–inflammation value

## Appendix A

**Table A1:** Subgroup comparison between confirmed and highly suspected *K. kingae* OAIs.

Parameter	Suspected Mean ± SD	Confirmed Mean ± SD	Wilcoxon *p*-Value
Abnormal CRP	40.32 ± 30.35	35.60 ± 24.19	0.398
ESR	35.74 ± 21.00	36.05 ± 18.57	0.815
Neutrophils	5.48 ± 2.57	5.57 ± 2.63	0.833
Lymphocytes	4.90 ± 2.27	5.16 ± 2.63	0.777
Monocytes	1.14 ± 1.33	1.53 ± 2.00	0.109
NLR	2.13 ± 3.40	1.84 ± 2.36	0.661
MLR	1.01 ± 3.61	0.99 ± 2.79	0.440
PLR	143.24 ± 205.64	127.33 ± 149.92	0.736
SII	794.74 ± 1230.37	746.85 ± 934.27	0.777
SIRI	5.65 ± 21.83	6.15 ± 17.42	0.562
PIV	2147.85 ± 8747.18	2560.24 ± 7807.42	0.501

Note: SD, standard deviation; CRP, C-reactive protein in mg/L; ESR, erythrocyte sedimentation rate in mm/h; NLR, neutrophil-to-lymphocyte ratio; MLR, monocyte-to-lymphocyte ratio; PLR, platelet-to-lymphocyte ratio; SII, systemic immune–inflammation index; SIRI, systemic inflammation response index; PIV, pan-immune–inflammation value.
